# Early Metabolic and Cardiovascular Alterations in Adolescents with MASLD or Simple Obesity

**DOI:** 10.3390/nu18213562

**Published:** 2026-10-28

**Authors:** Aldona Wierzbicka-Rucińska, Wojciech Jańczyk, Jacek Michałkiewicz, Łukasz Obrycki, Anna Świąder-Leśniak, Mieczysław Litwin, Zbigniew Kułaga, Jerzy Ostrowski, Piotr Socha

**Affiliations:** 1 Department of Clinical Biochemistry, The Children’s Memorial Health Institute, Aleja Dzieci Polskich 20, 04-730 Warsaw, Poland; 2 Deparment of Gastroenterology, Hepatology and Nutrition Disorders, The Children’s Memorial Health Institute, Aleja Dzieci Polskich 20, 04-730 Warsaw, Poland; w.janczyk@ipczd.pl (W.J.); p.socha@ipczd.pl (P.S.); 3 Department of Microbiology and Clinical Immunology, The Children’s Memorial Health Institute, Aleja Dzieci Polskich 20, 04-730 Warsaw, Poland; jjmichalkiewicz@wp.pl; 4 Department of Nephrology, Kidney Transplantation and Hypertension, The Children’s Memorial Health Institute, Aleja Dzieci Polskich 20, 04-730 Warsaw, Poland; l.obrycki@ipczd.pl (Ł.O.); m.litwin@ipczd.pl (M.L.); 5 Laboratory of Anthropology, The Children’s Memorial Health Institute, Aleja Dzieci Polskich 20, 04-730 Warsaw, Poland; a.swiader-lesniak@ipczd.pl; 6 Public Health Department of the Children’s Memorial Health Institute, Aleja Dzieci Polskich 20, 04-730 Warsaw, Poland; z.kulaga@ipczd.pl; 7 Department of Genetics, Maria Sklodowska-Curie National Research Institute of Oncology, 02-781 Warsaw, Poland; jostrow@warman.com.pl

**Keywords:** metabolic dysfunction-associated steatotic liver disease, pediatric obesity, controlled attenuation parameter, insulin resistance, TyG index, HOMA-IR, left ventricular mass index, pulse wave velocity, adolescents

## Abstract

**Background:** Hepatic steatosis is a frequent complication of adolescent obesity, but its association with metabolic and cardiovascular alterations beyond those related to obesity itself remains unclear. The primary objective of this study was to compare metabolic, hepatic, and cardiovascular characteristics among adolescents with obesity and hepatic steatosis (metabolic dysfunction-associated steatotic liver disease, MASLD), adolescents with obesity without hepatic steatosis, and healthy controls. The secondary, exploratory objective was to assess whether commonly used insulin resistance-related indices could discriminate MASLD from obesity without hepatic steatosis. **Methods:** This cross-sectional study included 111 adolescents aged 14–18 years: 50 with obesity and MASLD (controlled attenuation parameter [CAP] > 250 dB/m on transient elastography), 33 with obesity without hepatic steatosis (CAP ≤ 250 dB/m or inferred from normal weight status and absence of liver disease), and 28 healthy controls. CAP was available in 92 of 111 participants (49/50 MASLD, 22/33 obesity without steatosis, 21/28 controls). Assessments included anthropometry, bioimpedance-based body composition, liver enzymes, lipid profile, glucose–insulin metabolism, insulin resistance-related indices (HOMA-IR, TyG, VAI, TyG-BMI, and TyG-WC), pulse wave velocity (PWV), aortic blood pressure, carotid ultrasound, and echocardiography. **Results:** Compared with adolescents with obesity without steatosis, those with MASLD had higher CAP, alanine aminotransferase, gamma-glutamyl transferase, triglyceride levels, and waist circumference (*p* < 0.05), whereas surrogate insulin resistance indices did not differ significantly between the two groups. Compared with healthy controls, both clinical groups had higher left ventricular mass index and relative wall thickness (*p* < 0.05). PWV was higher in the MASLD group than in both controls (*p* = 0.030) and in adolescents with obesity without steatosis (*p* = 0.028), whereas carotid intima-media thickness did not differ significantly across groups. In receiver operating characteristic analyses, all evaluated insulin resistance-related indices showed poor discrimination between MASLD and obesity without hepatic steatosis, with AUC values ranging from 0.501 to 0.569. Waist circumference SDS was an important independent correlate of left ventricular mass index in regression analysis. **Conclusions:** Among adolescents with obesity, MASLD was associated with a less favorable hepatic and lipid profile and differences in selected cardiovascular measures. Waist circumference SDS was an important independent correlate of left ventricular mass index, highlighting the relevance of central adiposity in the assessment of cardiovascular characteristics. Commonly used surrogate insulin resistance-related indices showed poor ability to distinguish MASLD from obesity without hepatic steatosis and should not be used as standalone tools for this purpose. These findings support the complementary role of anthropometric, biochemical, cardiovascular, and noninvasive hepatic assessments in the evaluation of adolescents with obesity.

## 1. Introduction

Adolescent obesity represents a growing global public health challenge. According to the World Health Organization (WHO), the prevalence of overweight, including obesity, among children and adolescents aged 5–19 years increased from 8% in 1990 to 20% in 2022, while the prevalence of obesity rose from 2% to 8% over the same period [WHO]. Hepatic steatosis is one of the most common hepatic complications of pediatric obesity. A meta-analysis of 52 studies involving more than 15 million participants reported metabolic dysfunction-associated steatotic liver disease (MASLD) in 41.2% of children and adolescents with obesity, compared with 10.4% of those with overweight and 2.6% of those with normal weight, demonstrating a strong association between adiposity and hepatic steatosis [1]. Population-based transient elastography data from United States adolescents have also indicated a high prevalence of steatosis among those with obesity, although estimates vary according to the controlled attenuation parameter (CAP) threshold applied [2]. These findings highlight the importance of identifying adolescents at increased metabolic and hepatic risk, including those without overt clinical manifestations. Obesity in adolescence is associated with insulin resistance, dyslipidemia, elevated blood pressure, and hepatic steatosis [3,4,5]. These abnormalities reflect interconnected disturbances in glucose and lipid metabolism, insulin signaling, and low-grade inflammation that may contribute to MASLD development [6,7]. MASLD is increasingly recognized as a manifestation of systemic metabolic dysfunction rather than an isolated liver disorder. In addition to hepatic involvement, it has been associated with increased cardiometabolic risk, including arterial stiffness, endothelial dysfunction, and early subclinical cardiovascular alterations [8,9,10,11]. However, the extent to which these alterations are associated with hepatic steatosis independently of the degree of adiposity remains incompletely understood [12]. Assessing both metabolic and cardiovascular characteristics may therefore help characterize the early cardiometabolic phenotype of adolescents with obesity. The assessment of metabolic dysfunction in pediatric obesity remains challenging. Fasting glucose and insulin concentrations may not fully capture early disturbances in insulin sensitivity, while alanine aminotransferase (ALT), although widely used in clinical practice, has limited sensitivity for detecting hepatic steatosis in children and adolescents [11,13,14]. Several surrogate indices have consequently been investigated for metabolic risk assessment. The Homeostatic Model Assessment for Insulin Resistance (HOMA-IR) is widely used in clinical and research settings, but its interpretation in adolescents is affected by age, sex, pubertal stage, and population characteristics [12,15,16]. The triglyceride–glucose (TyG) index has also been proposed as a surrogate marker of insulin resistance. Composite indices combining metabolic and anthropometric measures, including TyG-BMI, TyG–waist circumference (TyG-WC), and the visceral adiposity index (VAI), have been developed to capture aspects of the relationship between adiposity and metabolic dysfunction [17,18,19,20]. Some pediatric studies have reported promising performance of these indices for identifying hepatic steatosis, with area under the receiver operating characteristic curve (AUC) values exceeding 0.75 [1,19,21]. However, their performance may vary across populations and diagnostic definitions. Their ability to distinguish adolescents with MASLD from those with obesity but without hepatic steatosis is particularly relevant clinically, because both groups share an important underlying risk factor: excess adiposity. Furthermore, puberty-related changes in insulin sensitivity, somatic growth, and body composition may affect the relationships between these indices and metabolic dysfunction. Many of these measures were initially developed or extensively evaluated in adults, and their diagnostic utility in pediatric populations remains insufficiently established [12,15,16]. The diagnosis of MASLD requires evidence of hepatic steatosis in the context of cardiometabolic risk. Nevertheless, the assessment of steatosis in children and adolescents remains challenging because noninvasive diagnostic methods and thresholds may perform differently across populations [10,11,21,22]. In the present study, hepatic steatosis was assessed using CAP, with a threshold of >250 dB/m used to classify participants as having steatosis. Other CAP thresholds, including 248, 263, and 290 dB/m, have been proposed in pediatric studies, reflecting a trade-off between sensitivity and specificity. Lower thresholds may identify more cases of mild steatosis but can reduce specificity, whereas higher thresholds may improve specificity while missing some cases of early disease. No single threshold has been universally validated for all pediatric populations [2,23]. The selected threshold is therefore important when interpreting group differences and evaluating the performance of metabolic indices. Obesity and hepatic steatosis may also be associated with early cardiovascular remodeling, including increased left ventricular mass, changes in ventricular geometry, and altered vascular function [9,24]. Visceral adiposity may contribute to these changes through interconnected metabolic, inflammatory, and hemodynamic pathways [9,10,24]. However, it remains uncertain whether hepatic steatosis provides additional information about cardiovascular alterations beyond that associated with obesity itself. Examining hepatic, metabolic, and cardiovascular characteristics together may therefore provide a more comprehensive description of early cardiometabolic risk in adolescents. Against this background, the primary objective of this study was to compare metabolic and cardiovascular characteristics among adolescents with obesity and MASLD, adolescents with obesity without hepatic steatosis, and healthy controls. The principal comparison was between the two groups with obesity, with the control group providing a reference for differences associated with the broader clinical phenotype. The study assessed anthropometric and body composition measures, fasting glucose and insulin, HOMA-IR, TyG, VAI, TyG-BMI, TyG-WC, lipid profile, liver-related parameters, and cardiovascular structure and function [17,18,19,23]. A secondary, exploratory objective was to evaluate the ability of selected insulin resistance-related indices to distinguish adolescents with MASLD from those with obesity without hepatic steatosis. We hypothesized that adolescents with MASLD would exhibit a less favorable metabolic and cardiovascular profile than healthy controls and would differ from adolescents with obesity without steatosis in selected clinical and biochemical characteristics. We also explored whether insulin resistance-related indices could discriminate between the two groups with obesity, while recognizing that their performance in adolescents might be limited. Importantly, because all participants in the two clinical groups had obesity, the study design permits comparison of adolescents who differ in the presence of hepatic steatosis but does not allow estimation of a steatosis-associated effect that is fully independent of adiposity severity.

## 2. Materials and Methods

### 2.1. Study Population and Design

**Figure 1:**
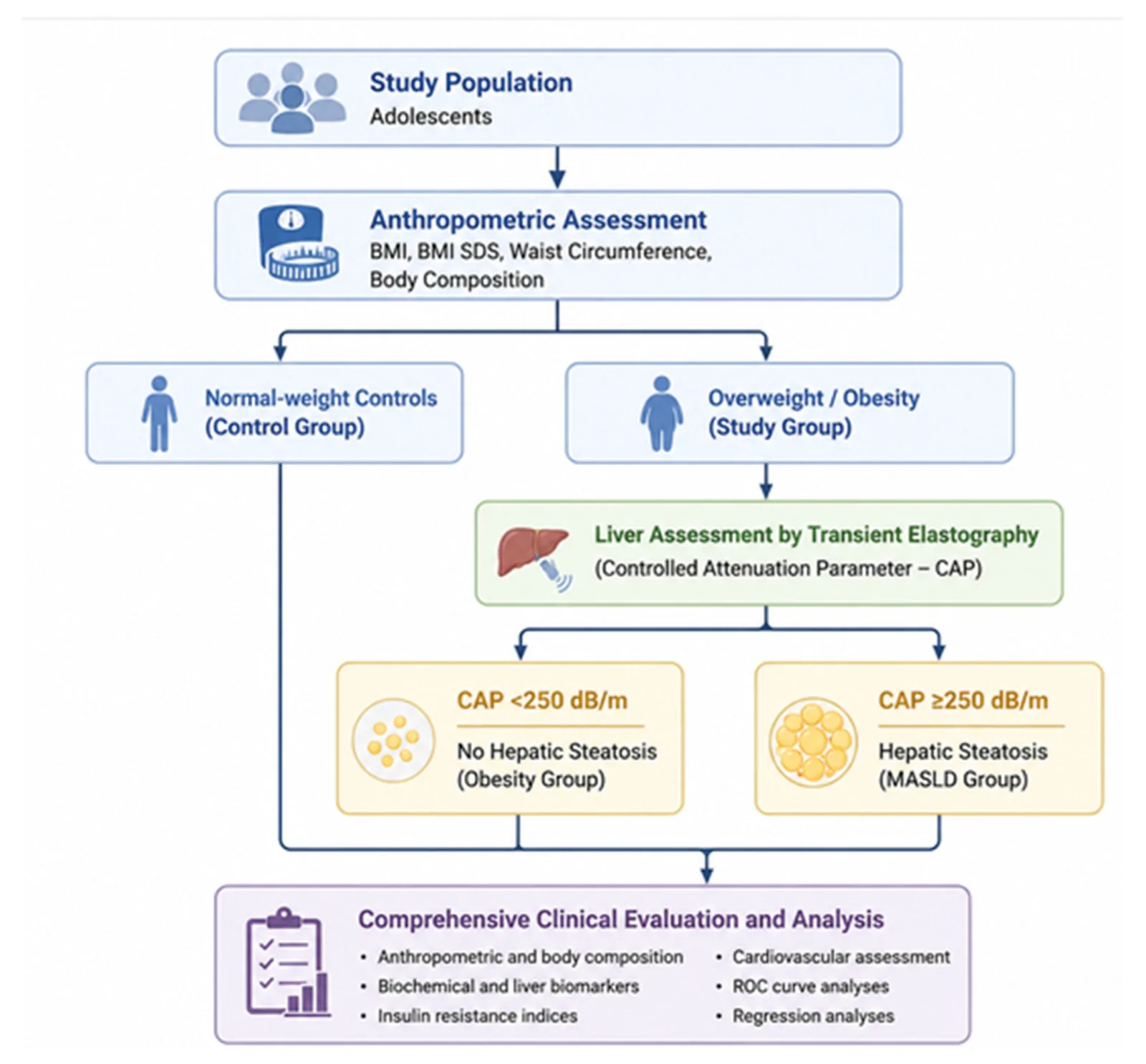
Study design and participant stratification according to body mass index (BMI) and controlled attenuation parameter (CAP).

This cross-sectional study included adolescents aged 14–18 years who were recruited at the Children’s Memorial Health Institute, Warsaw, Poland, between January 2021 and May 2023. Participants were classified into three groups: (1) adolescents with obesity and metabolic dysfunction-associated steatotic liver disease (MASLD), (2) adolescents with obesity without hepatic steatosis, and (3) healthy normal-weight controls. Obesity was defined according to age- and sex-specific body mass index standard deviation scores (BMI SDS) based on International Obesity Task Force (IOTF) criteria. Among participants with obesity, hepatic steatosis was classified primarily using the controlled attenuation parameter (CAP) obtained by vibration-controlled transient elastography (VCTE). Participants with CAP values > 250 dB/m were assigned to the MASLD group, whereas those with CAP values ≤ 250 dB/m were classified as having obesity without hepatic steatosis. Healthy normal-weight adolescents served as controls. Controlled attenuation parameter (CAP) measurements were obtained from all participants with obesity (*n* = 71) as part of the hepatic steatosis diagnostic protocol. Among healthy controls (*n* = 28), CAP was successfully measured in 21 participants (75.0%). Seven control participants did not undergo transient elastography due to technical constraints during the study period. For these seven participants, absence of hepatic steatosis was inferred based on normal weight status and absence of clinical evidence of liver disease. Additionally, among adolescents with obesity without steatosis, CAP was available in only 22 of 33 participants; the remaining 11 were classified based on normal liver enzymes, normal liver stiffness where available, and absence of clinical evidence of liver disease. This differential availability of CAP measurements between groups constitutes a study limitation acknowledged in Section 6. Accordingly, hepatic steatosis was not excluded by CAP measurement in every control participant. Exclusion criteria included chronic liver diseases other than MASLD, viral hepatitis B or C, autoimmune liver disease, known genetic or metabolic liver diseases (including Wilson’s disease, α1-antitrypsin deficiency, and hemochromatosis), chronic treatment with medications known to promote hepatic steatosis, and severe chronic diseases. Viral hepatitis screening included hepatitis B surface antigen (HBsAg) and antibodies against hepatitis C virus (anti-HCV). Alcohol consumption was assessed by interview with the participant and caregiver; no participant reported regular alcohol intake. The study flow and participant classification are presented in Figure 1.

### 2.2. Hepatic Steatosis and Liver Stiffness Assessment

Hepatic steatosis and liver stiffness were assessed using VCTE (FibroScan^®^ 502 Touch, Echosens, Paris, France). Examinations were performed by trained operators after a fasting period of 2–3 h. This interval applied to elastography and was distinct from the overnight fasting period of 10–12 h used before blood sampling. The M probe was used as the default probe, whereas the XL probe was selected according to the skin-to-liver capsule distance, particularly in participants with obesity. Among the 78 participants with valid CAP measurements, the XL probe was used in 32 (41.0%) and the M probe in 46 (59.0%). Probe type was recorded for each examination. At least 10 valid measurements were obtained for each examination. Measurement reliability was assessed using established quality criteria, including an interquartile range-to-median ratio (IQR/M) ≤ 30% and a success rate ≥ 60%. Liver stiffness was expressed in kilopascals (kPa) and reported as the median of valid measurements. Hepatic steatosis was defined as CAP > 250 dB/m, a threshold previously applied in pediatric studies [23]. Because CAP measurements may vary according to probe type, the use of M and XL probes was considered when interpreting CAP-based classification. The influence of probe type was evaluated in a sensitivity analysis where the available data permitted.

### 2.3. Anthropometric and Clinical Measurements

Anthropometric measurements were performed by trained personnel using standardized procedures. Body weight and height were measured in duplicate. BMI was calculated as weight in kilograms divided by height in meters squared. BMI SDS was calculated using age- and sex-specific reference data. Waist and hip circumferences were measured using standardized anatomical landmarks. Body composition, including fat mass and lean body mass, was assessed by multifrequency bioelectrical impedance analysis (BIA; Tanita MC-780MA, Tanita Corporation, Tokyo, Japan). BIA was selected because it is non-invasive, rapid, and suitable for body-composition assessment in the clinical setting without exposure to ionizing radiation. Dual-energy X-ray absorptiometry (DXA) was not routinely available within the study protocol.

Adolescents with obesity underwent hepatic assessment by vibration-controlled transient elastography, and hepatic steatosis was classified according to CAP values. A CAP value > 250 dB/m was used to define the hepatic steatosis group (MASLD), whereas CAP ≤ 250 dB/m was used to classify participants as having obesity without hepatic steatosis. The study subsequently evaluated anthropometric, body-composition, biochemical, insulin resistance-related, and cardiovascular parameters, together with ROC and regression analyses. The flowchart illustrates the analytical framework used to investigate whether hepatic steatosis was associated with an adverse metabolic and cardiovascular phenotype and whether surrogate insulin resistance indices could distinguish MASLD from obesity without hepatic steatosis.

### 2.4. Cardiovascular Assessment

Blood pressure was measured using a validated oscillometric device after a 5 min rest period. Measurements were obtained during at least three separate visits, and mean values were used for analysis. Arterial stiffness and central hemodynamic parameters were assessed using the Vicorder^®^ system (SMT Medical GmbH, Würzburg, Germany). Carotid intima-media thickness (cIMT) and carotid wall cross-sectional area (WCSA) were evaluated by high-resolution ultrasonography. Transthoracic echocardiography was performed to assess left ventricular mass index (LVMi) and relative wall thickness (RWT).

### 2.5. Laboratory Analyses

Venous blood samples were collected after an overnight fast of 10–12 h. Serum was separated by centrifugation at 4000 rpm for 10 min. Samples were analyzed immediately or stored at 0–8 °C for up to 48 h or at −70 °C until analysis. All laboratory analyses were performed at the Department of Clinical Biochemistry, Children’s Memorial Health Institute. Fasting glucose, lipid profile, liver enzymes, and other routine biochemical parameters were measured using standard enzymatic methods on a Siemens biochemical analyzer. Serum insulin concentrations were determined using a chemiluminescent immunoassay (Siemens Atellica).

### 2.6. Calculation of Insulin Resistance and Adiposity Indices

Insulin resistance and metabolic dysfunction were assessed using surrogate indices derived from fasting biochemical and anthropometric measurements. Glucose and triglyceride concentrations were expressed in mg/dL.

The Homeostatic Model Assessment for Insulin Resistance (HOMA-IR) was calculated as follows [12]:

HOMA-IR = [fasting glucose (mg/dL) × fasting insulin (µU/mL)]/405

The triglyceride–glucose (TyG) index was calculated using the standard equation [17]:

TyG = ln [(fasting triglycerides (mg/dL) × fasting plasma glucose (mg/dL))/2]

The visceral adiposity index (VAI) was calculated using the original sex-specific equations:

Males: VAI = [WC/(39.68 + 1.88 × BMI)] × (TG/1.03) × (1.31/HDL-C)

Females: VAI = [WC/(36.58 + 1.89 × BMI)] × (TG/0.81) × (1.52/HDL-C)

Composite indices were calculated as follows [25]:

TyG-BMI = TyG × BMI

TyG-WC = TyG × waist circumference

Because VAI, TyG-BMI, and TyG-WC have been validated primarily in adult populations, these indices were included as exploratory surrogate markers of insulin resistance and adiposity in this adolescent cohort. Their interpretation therefore focused on between-group comparisons rather than on the clinical significance of absolute values [23].

### 2.7. Analytical Approach

The primary analyses compared adolescents with MASLD, adolescents with obesity without hepatic steatosis, and healthy normal-weight controls. Additional analyses evaluated associations between insulin resistance-related indices and anthropometric, biochemical, hepatic, and inflammatory parameters. Regression analyses were used to investigate factors associated with LVMi, and receiver operating characteristic (ROC) analyses were performed to assess the ability of surrogate indices to discriminate adolescents with MASLD from those with obesity without hepatic steatosis. Sex-stratified analyses were performed for selected anthropometric and cardiovascular parameters to explore potential differences between female and male participants. These subgroup analyses were considered exploratory.

### 2.8. Statistical Analysis

Statistical analyses were performed using SAS version 9.4 (SAS Institute Inc., Cary, NC, USA). The distribution of continuous variables was assessed using the Kolmogorov–Smirnov test and graphical inspection. Continuous data are presented as mean ± standard deviation (SD) or median and interquartile range (IQR), as appropriate. Comparisons among the three study groups were performed using one-way analysis of variance (ANOVA), followed by Tukey’s post hoc test, for variables meeting the assumptions of normality and homogeneity of variance. For variables that did not meet the assumptions for parametric analysis, the Kruskal–Wallis test followed by Dunn’s post hoc test was used. The assumptions underlying parametric analyses were assessed before the relevant tests were applied. Associations between insulin resistance-related indices and clinical variables were evaluated using Pearson or Spearman correlation coefficients, with the method selected according to the distributional characteristics of the variables. A total of 85 correlations were evaluated. Nominal *p* values are reported, and multiple testing was addressed using the Benjamini–Hochberg false discovery rate (FDR) procedure applied across all 85 correlation tests. A Bonferroni-adjusted threshold of 0.05/85 (approximately 0.00059) was also considered as a conservative reference. Univariable and multivariable linear regression analyses were performed to investigate factors associated with left ventricular mass index (LVMi). Candidate predictors included anthropometric, body-composition, and biochemical parameters. Because several adiposity measures are interrelated, waist circumference SDS was retained a priori as the single representative measure of central adiposity in the multivariable model to limit multicollinearity. Variables with *p* < 0.10 in univariable analysis were entered into the multivariable model, and variables with *p* < 0.05 were retained in the final model. Multicollinearity was assessed using variance inflation factors (VIFs); all VIF values in the final model were below <0,05. Model assumptions were evaluated by residual diagnostics, including assessment of residual normality (Shapiro–Wilk test and Q–Q plots), homoscedasticity (residuals versus fitted values plot), and influential observations (Cook’s distance). Receiver operating characteristic (ROC) curve analysis was used to assess the ability of HOMA-IR, TyG, VAI, TyG-BMI, and TyG-WC to discriminate adolescents with MASLD from those with obesity without hepatic steatosis. Discriminatory performance was summarized using the area under the ROC curve (AUC). Missing observations were handled by complete-case analysis for each variable; no imputation was performed. The number of participants contributing data to each analysis is reported in the corresponding tables. All statistical tests were two-sided. A *p* value < 0.05 was considered statistically significant for unadjusted analyses, while FDR-adjusted results were interpreted according to the Benjamini–Hochberg procedure.

### 2.9. Ethical Considerations

The study protocol was approved by the Ethics Committee of the Children’s Memorial Health Institute (approval no. 34/KBE/2019). Written informed consent was obtained from participants and their parents or legal guardians in accordance with the approved study protocol and applicable requirements for minors. All study procedures were conducted in accordance with the principles of the Declaration of Helsinki.

## 3. Results

### 3.1. Anthropometric Characteristics and Body Composition

A total of 111 adolescents were included in the final analysis: 50 with metabolic dysfunction-associated steatotic liver disease (MASLD; hepatic steatosis defined as a controlled attenuation parameter [CAP] value > 250 dB/m), 33 with obesity without hepatic steatosis, and 28 healthy controls. Group assignment was based primarily on the CAP value. Participants with overweight or obesity and a CAP value > 250 dB/m were classified as having MASLD, irrespective of their initial clinical referral diagnosis. Anthropometric and body-composition characteristics are presented in Table 1. The three groups did not differ significantly in age (*p* = 0.170). Compared with healthy controls, both clinical groups had higher body weight, BMI, waist circumference, and measures of adiposity. Comparisons between the two clinical groups were of particular interest because they allowed the assessment of anthropometric and body-composition differences associated with hepatic steatosis among adolescents with obesity. The primary comparison was between adolescents with MASLD and those with obesity without hepatic steatosis. Comparisons with healthy controls provided a secondary reference for evaluating differences associated with obesity and metabolic health. Because the clinical groups could differ in the severity and distribution of adiposity, between-group differences in metabolic and cardiovascular parameters were interpreted with consideration of adiposity and sex, as described in Section 2.7 and Section 2.8.

### 3.2. Sex-Specific Anthropometric Characteristics

Anthropometric analyses were additionally performed separately for female and male participants because body composition differs substantially by sex during adolescence. The results are presented in Table 2 and Table 3. Among females, age and height did not differ significantly between the study groups. Compared with healthy controls, girls in both clinical groups had higher body weight, body weight SDS, BMI, BMI z-score, and BMI SDS. Compared with girls with obesity without hepatic steatosis, girls with MASLD had significantly higher body weight and body weight SDS (*p* = 0.010 and *p* = 0.018, respectively), whereas BMI and BMI-derived indices did not differ significantly between the two clinical groups. Among males, age and height SDS were comparable across groups, although boys with MASLD were slightly taller than those with obesity without hepatic steatosis (*p* = 0.043). Both clinical groups had higher body weight, body weight SDS, BMI, BMI z-score, BMI SDS, waist circumference, waist circumference SDS, and fat mass than healthy controls. Lean body mass was significantly greater in boys with MASLD than in healthy controls (*p* < 0.001), whereas no significant difference was observed between boys with obesity and controls. Compared with boys with obesity without hepatic steatosis, those with MASLD had significantly higher body weight, waist circumference, waist circumference SDS, lean body mass, and fat mass, while BMI-derived indices and body fat percentage remained comparable. Overall, sex-stratified analyses suggested that differences between adolescents with MASLD and those with obesity without hepatic steatosis were more apparent among males, particularly for measures of central adiposity and body composition. These findings should be interpreted cautiously because of the relatively small sample sizes in the sex-specific subgroups.

### 3.3. Biochemical and Elastography Parameters

Biochemical and liver elastography findings are summarized in Table 4. Liver stiffness (E, kPa) did not differ significantly among the three groups (overall *p* = 0.82; all pairwise *p* ≥ 0.54). CAP differed significantly among groups (overall *p* < 0.001), with the highest values in adolescents with MASLD (median 293.0 dB/m) and lower values in those with obesity without hepatic steatosis (222.0 dB/m) and healthy controls (189.0 dB/m). CAP was significantly higher in the MASLD group than in both comparison groups (both *p* < 0.001), whereas the difference between the obesity group and controls did not reach statistical significance (*p* = 0.158). Markers of hepatocellular injury showed a similar pattern. ALT was significantly higher in adolescents with MASLD (median 33.5 U/L) than in those with obesity without hepatic steatosis (20.0 U/L; *p* = 0.018) and healthy controls (12.0 U/L; *p* < 0.001); ALT was also higher in the obesity group than in controls (*p* = 0.0015). GGT was higher in the MASLD group (23.0 U/L) than in the obesity group (16.0 U/L; *p* = 0.0049) and controls (16.0 U/L; *p* = 0.0078), whereas the latter two groups did not differ (*p* = 0.951). AST differed significantly among groups (overall *p* < 0.001), being higher in the MASLD group (23.0 U/L) than in controls (14.5 U/L; *p* < 0.001), with no significant difference between the MASLD and obesity groups (*p* = 0.072); AST was also higher in the obesity group than in controls (*p* = 0.013). Total bilirubin and C-reactive protein (CRP) did not differ significantly among groups (overall *p* = 0.11 and *p* = 0.35, respectively). Among lipid parameters, triglycerides showed the most pronounced between-group differences (overall *p* < 0.001), with the highest concentrations in the MASLD group (median 126.0 mg/dL), intermediate concentrations in the obesity group (92.0 mg/dL), and the lowest concentrations in controls (66.5 mg/dL). Triglycerides were significantly higher in the MASLD group than in both comparison groups (MASLD vs. obesity *p* = 0.046; MASLD vs. controls *p* < 0.001), and higher in the obesity group than in controls (*p* = 0.0020). HDL-C was significantly lower in both clinical groups (MASLD 40.0 mg/dL; obesity 41.0 mg/dL) than in healthy controls (54.0 mg/dL; both *p* < 0.001), whereas the two clinical groups did not differ (*p* = 0.664). Total cholesterol and LDL-C did not differ significantly among groups (overall *p* = 0.57 and *p* = 0.75, respectively). Fasting glucose concentrations were comparable across groups (overall *p* = 0.25; all pairwise *p* ≥ 0.097). Fasting insulin was significantly higher in adolescents with MASLD (median 15.30 µIU/mL) than in healthy controls (9.35 µIU/mL; *p* = 0.0003), whereas the difference between the MASLD and obesity groups (11.95 µIU/mL; *p* = 0.090) and between the obesity and control groups (*p* = 0.088) did not reach statistical significance. All five surrogate indices of insulin resistance and adiposity differed significantly across the three groups (overall *p* < 0.001 for each; Table 4). In each case the gradient was ordered MASLD > obesity without steatosis > controls: HOMA-IR (medians 3.40, 2.84, and 2.01), TyG index (8.68, 8.40, and 7.95), VAI (1.94, 1.51, and 0.75), TyG-BMI (271.5, 236.9, and 156.4), and TyG-WC (826.5, 742.2, and 553.7). For four of the five indices, both clinical groups differed significantly from healthy controls (TyG: *p* = 0.0018 and *p* < 0.001; VAI: *p* = 0.0002 and *p* < 0.001; TyG-BMI and TyG-WC: both *p* < 0.001). HOMA-IR differed significantly between the MASLD group and controls (*p* = 0.0001) but not between the obesity group and controls (*p* = 0.069). In contrast, none of the four conventional indices differed significantly between adolescents with MASLD and those with obesity without hepatic steatosis (HOMA-IR *p* = 0.085; TyG *p* = 0.144; VAI *p* = 0.061; TyG-BMI *p* = 0.113), and the difference in TyG-WC was borderline (*p* = 0.049). These gradients are consistent with the strong correlations between the indices and anthropometric measures reported in Table. Overall, CAP, ALT, GGT, triglyceride concentrations, and HDL-C most clearly distinguished adolescents with MASLD from healthy controls and, for several parameters, from adolescents with obesity without hepatic steatosis. The surrogate indices of insulin resistance and adiposity differed significantly across the three groups, but this reflected primarily their separation from healthy controls; their ability to discriminate MASLD from obesity without hepatic steatosis was limited (Section 3.7), consistent with the substantial overlap in metabolic profile between these two groups, which share excess adiposity as a common underlying risk factor.

### 3.4. Cardiovascular and Vascular Parameters

Cardiovascular and vascular parameters are summarized in Table 5. Throughout this section, increased pulse wave velocity (PWV), left ventricular mass index (LVMi), and relative wall thickness (RWT) are interpreted as subclinical cardiovascular alterations and early cardiac remodeling rather than as evidence of established cardiovascular disease. PWV was significantly higher in adolescents with MASLD than in healthy controls (*p* = 0.030). The pairwise comparison between the MASLD and obesity groups was also significant (*p* = 0.028), whereas no significant difference was observed between the obesity and control groups. Peripheral systolic and diastolic blood pressure did not differ significantly among groups, and the proportion of participants with aortic blood pressure (AoBP) above the 95th percentile was comparable across groups. Central AoBP was slightly higher in adolescents with MASLD than in healthy controls (*p* = 0.049), with no significant differences between the MASLD and obesity groups or between the obesity and control groups. Because PWV is influenced by blood pressure, the observed differences were interpreted cautiously as potential early subclinical vascular alterations rather than evidence of established arterial disease. Carotid structural parameters showed limited between-group differences. Right and left carotid intima-media thickness (IMTR and IMTL, respectively) and mean common carotid intima-media thickness (cIMT) were comparable among groups. In contrast, wall cross-sectional area (WCSA) was significantly higher in adolescents with MASLD than in healthy controls (*p* = 0.002), whereas the other pairwise comparisons were not significant. Because absolute WCSA may be influenced by body size and carotid geometry, this finding warrants cautious interpretation. The combination of higher WCSA and unchanged cIMT may be compatible with early vascular wall remodeling, although the present cross-sectional findings do not establish the underlying mechanism. Cardiac structural parameters differed significantly among groups. LVMi was significantly higher in both the MASLD group (*p* < 0.001) and the obesity group (*p* = 0.001) than in healthy controls, with no significant difference between the two clinical groups. Similarly, RWT was significantly greater in adolescents with MASLD (*p* = 0.008) and those with obesity without hepatic steatosis (*p* = 0.015) than in controls, whereas the two clinical groups did not differ significantly. Taken together, these findings indicate that early subclinical cardiac remodeling was associated primarily with obesity and adiposity, rather than with hepatic steatosis independently of the degree of adiposity. Vascular differences were less consistent and were most apparent for PWV and WCSA.

Sex-specific cardiovascular analysis. Sex-stratified cardiovascular analyses, performed as sensitivity analyses, are presented in Table 6. Among females, no significant between-group differences were observed in PWV, peripheral or central blood pressure, carotid parameters, WCSA, or RWT. LVMi was significantly higher in girls with MASLD (*p* = 0.020) and girls with obesity (*p* = 0.023) than in healthy controls, with no significant difference between the two clinical groups. Among males, PWV was significantly higher in adolescents with MASLD than in healthy controls (*p* = 0.030), whereas the difference between the MASLD and obesity groups was not significant. Central AoBP was slightly higher in boys with MASLD than in controls (*p* = 0.049), and WCSA was significantly greater in the MASLD group than in controls (*p* = 0.002). IMTR, IMTL, and cIMT did not differ significantly among the male subgroups. Cardiac structural differences were also evident among males. LVMi was significantly higher in boys with MASLD (*p* = 0.007) and boys with obesity (*p* = 0.001) than in healthy controls. RWT was significantly greater in both the MASLD (*p* = 0.003) and obesity (*p* = 0.027) groups than in controls, with no significant difference between the two clinical groups. Overall, the sex-stratified analyses were broadly consistent with the findings in the full cohort. Subclinical cardiac remodeling, reflected by higher LVMi and RWT, was observed in both sexes, whereas vascular differences were more apparent among males. These findings should be interpreted cautiously because of the relatively small sample sizes in the sex-specific subgroups.

### 3.5. Correlations Between Insulin Resistance Indices and Clinical Parameters

Correlation analyses are summarized in Table 7. Overall, the evaluated indices showed moderate-to-strong correlations with anthropometric and metabolic parameters. HOMA-IR correlated strongly with fasting insulin (*r* = 0.987, *p* < 0.001) and moderately with measures of adiposity, including fat mass (*r* = 0.448), waist circumference (*r* = 0.410), body weight (*r* = 0.471), and BMI (*r* = 0.385) (all *p* < 0.001). The TyG index correlated strongly with triglycerides (*r* = 0.907, *p* < 0.001), moderately with waist circumference (*r* = 0.606, *p* < 0.001), and inversely with HDL-C (*r* = −0.482, *p* < 0.001). The composite indices showed the expected strong correlations with the anthropometric components from which they are derived: TyG-BMI with BMI (*r* = 0.972) and TyG-WC with waist circumference (*r* = 0.963) (both *p* < 0.001). HOMA-IR was additionally associated with triglycerides (*r* = 0.323, *p* = 0.004), ALT (*r* = 0.326, *p* = 0.005), AST (*r* = 0.236, *p* = 0.045), and inversely with HDL-C (*r* = −0.264, *p* = 0.023). Liver enzymes correlated significantly with most indices, most consistently with the TyG index (ALT *r* = 0.374, AST *r* = 0.402, GGT *r* = 0.393; all *p* ≤ 0.001 after FDR adjustment). No significant correlations were observed between any of the indices and age or CRP. Given the large number of comparisons (*n* = 85), nominal *p* values are reported for transparency, and statistical significance was assessed using the Benjamini–Hochberg false discovery rate procedure; correlations with *p* < 0.001 remained significant after FDR adjustment. Overall, the observed correlations were dominated by the shared anthropometric and biochemical components of the indices and involved not only measures of adiposity but also lipid parameters and liver enzymes. These strong associations reflect, at least in part, the mathematical construction of the indices, and their magnitude should not be interpreted as evidence of discriminative ability between diagnostic groups—a question addressed directly by the ROC analyses (Section 3.7).

### 3.6. Determinants of Left Ventricular Remodeling

Univariate and multivariable regression analyses of factors associated with left ventricular mass index (LVMi) are presented in Table 8. In univariate analyses, LVMi was positively associated with BMI SDS (β = 28.80, *p* < 0.0001), waist circumference SDS (β = 27.23, *p* < 0.0001), lean body mass (β = 2.87, *p* < 0.0001), body fat mass (β = 2.87, *p* < 0.0001), body fat percentage (β = 3.16, *p* < 0.0001), total cholesterol (β = 0.41, *p* = 0.0318), triglycerides (β = 0.44, *p* < 0.0001), HOMA-IR (β = 9.29, *p* = 0.020), the TyG index (β = 0.010, *p* < 0.0001), and TyG-BMI (β = 0.260, *p* = 0.0002), and inversely with HDL-C (β = −2.44, *p* = 0.0001). Because of the marked collinearity among adiposity measures (BMI SDS, waist circumference SDS, lean body mass, body fat mass, and body fat percentage), waist circumference SDS was retained as the single representative measure of central adiposity to limit multicollinearity; variance inflation factors for the final model were verified (all VIF < 2.5). In the final multivariable model, waist circumference SDS remained the strongest independent determinant of LVMi (β = 21.37, *p* < 0.0001). Total cholesterol remained independently associated with higher LVMi (β = 0.45, *p* = 0.0046), and HDL-C with lower LVMi (β = −1.74, *p* = 0.0030). Model assumptions were verified by residual diagnostics (normality of residuals, homoscedasticity, absence of influential observations). Overall, the multivariable analysis identified central adiposity, together with total and HDL-cholesterol concentrations, as independent determinants of LVMi in the studied adolescents, supporting the concept that cardiac remodeling in obese adolescents accompanies obesity severity—particularly central fat distribution—rather than hepatic steatosis beyond the degree of adiposity.

### 3.7. Diagnostic Performance of Insulin Resistance Indices

**Figure 2:**
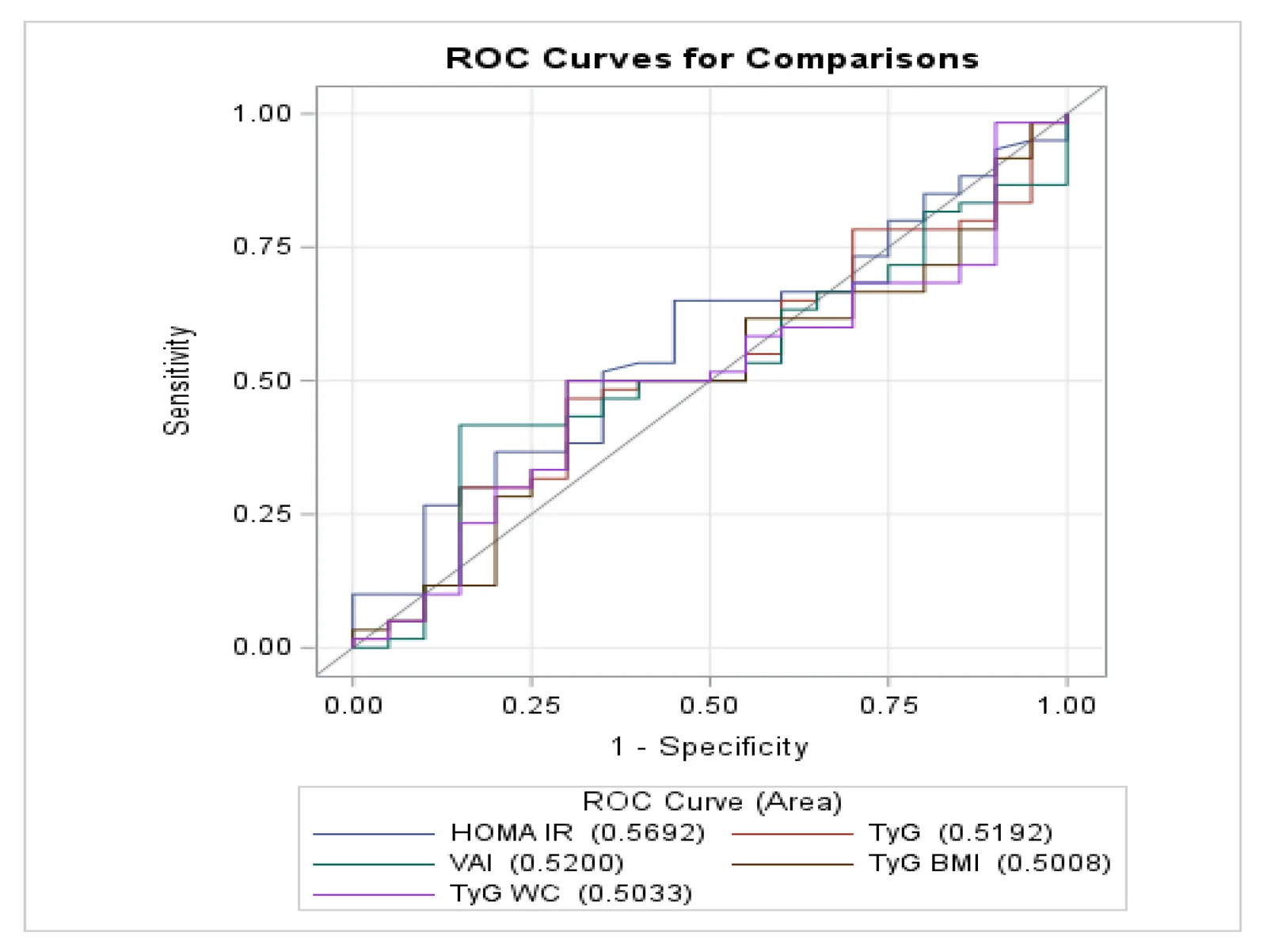
Receiver operating characteristic (ROC) curves of HOMA-IR, the TyG index, TyG-BMI, TyG-WC, and VAI for discriminating adolescents with MASLD from obese adolescents without hepatic steatosis. The diagonal line represents discrimination no better than chance (AUC = 0.50). All indices showed poor discriminatory performance (AUC 0.501–0.569), with curves closely approximating the diagonal, indicating that none of these surrogate indices can reliably identify hepatic steatosis among adolescents with established obesity. Direct assessment of hepatic steatosis (e.g., CAP during transient elastography) therefore remains necessary in this population.

Receiver operating characteristic (ROC) curve analysis was performed to evaluate the ability of insulin resistance-related indices to discriminate adolescents with MASLD from adolescents with obesity without hepatic steatosis (Figure 2). Overall, all evaluated indices showed poor discriminatory performance, with area under the curve (AUC) values ranging from 0.501 to 0.569. HOMA-IR yielded the highest AUC (0.569), although its discriminatory ability remained limited. The visceral adiposity index (VAI) yielded an AUC of 0.520, while the TyG index, TyG-BMI, and TyG-WC yielded AUC values of 0.519, 0.501, and 0.503, respectively. These results indicate that none of the evaluated insulin resistance-related indices reliably discriminated adolescents with MASLD from those with obesity without hepatic steatosis in this cohort. The findings should be interpreted cautiously because of the exploratory nature of the analysis and the relatively small sample size. Direct assessment of hepatic steatosis, such as controlled attenuation parameter (CAP) measurement during transient elastography, therefore remains important when evaluating hepatic steatosis in this population.

## 4. Discussion

The present study provides an integrated assessment of anthropometric, metabolic, hepatic, and cardiovascular characteristics in adolescents with obesity, stratified according to the presence of metabolic dysfunction-associated steatotic liver disease (MASLD). Several findings emerged. First, adolescents with MASLD exhibited a more pronounced adiposity phenotype than those with obesity without hepatic steatosis. Second, MASLD was associated with a less favorable biochemical profile, particularly higher liver enzyme activities and triglyceride concentrations and lower HDL-cholesterol levels than in healthy controls. Third, left ventricular mass index (LVMi) and relative wall thickness (RWT) were higher in both clinical groups than in controls, while multivariable regression identified waist circumference standard deviation score (SDS) as an important independent correlate of LVMi. Finally, the evaluated surrogate indices of insulin resistance showed poor discriminatory performance for distinguishing adolescents with MASLD from those with obesity without steatosis. Collectively, these findings support the view that pediatric MASLD occurs within a broader cardiometabolic context characterized by excess adiposity and associated metabolic disturbances [6,7,9,10]. In the present cohort, adolescents with MASLD had greater body weight, waist circumference, and fat mass than adolescents with obesity without hepatic steatosis, despite relatively small differences in BMI SDS. This pattern suggests that the distribution and composition of adiposity may provide complementary information to BMI when characterizing adolescents at risk of hepatic steatosis. Waist circumference is a practical surrogate marker of central adiposity, although it does not directly quantify visceral fat. Increased free fatty acid delivery to the liver and associated disturbances in lipid metabolism have been proposed as mechanisms linking central adiposity to hepatic fat accumulation and systemic metabolic dysfunction [7,16]. The present findings are consistent with previous pediatric studies emphasizing the relevance of abdominal adiposity to MASLD beyond conventional BMI-based assessment [7,10,11,25,26]. However, because the study was cross-sectional, the observed differences cannot establish whether central adiposity precedes or directly causes hepatic steatosis. Biochemically, adolescents with MASLD had higher ALT and GGT activities than the comparison groups, whereas liver stiffness did not differ significantly between groups. This combination of increased CAP and higher liver enzyme activities, without detectable differences in liver stiffness, is compatible with a phenotype in which steatosis and biochemical evidence of hepatocellular injury are present without demonstrable differences in liver stiffness [3,6,10,11,13,14]. Nevertheless, normal or comparable liver stiffness measurements do not exclude early fibrosis or other histological abnormalities, and transient elastography cannot replace liver biopsy when histological assessment is clinically indicated. CAP and liver stiffness provide complementary information, and their findings should be interpreted within the limitations of noninvasive assessment in pediatric populations [11,13,14,23]. Dyslipidemia was another prominent feature of the MASLD group. Triglyceride concentrations were higher in adolescents with MASLD than in both comparison groups, while HDL-C concentrations were lower than in healthy controls. The triglyceride difference between adolescents with MASLD and those with obesity without steatosis is particularly relevant because it suggests that lipid disturbances may help characterize metabolic differences within an already obese population. Hepatic steatosis has been linked in previous studies to altered very-low-density lipoprotein (VLDL) metabolism, increased hepatic triglyceride availability, and impaired lipid handling [7,16]. These mechanisms were not directly assessed in the present study and therefore remain potential explanations rather than demonstrated pathways. Although HDL-C was inversely associated with LVMi in the multivariable analysis, this association does not establish that dyslipidemia mediates a causal relationship between MASLD and cardiac remodeling. Previous pediatric studies have likewise identified elevated triglycerides and reduced HDL-C as important components of the cardiometabolic profile associated with MASLD [9,10,16,26]. Despite differences in hepatic steatosis and lipid parameters, fasting glucose concentrations were comparable across groups, and the differences in fasting insulin and surrogate indices of insulin resistance were limited. Neither HOMA-IR nor the evaluated TyG-derived indices significantly differentiated the clinical groups. This finding suggests that these indices may have limited discriminatory value when used to identify hepatic steatosis among adolescents who already have obesity. Insulin resistance is common in pediatric obesity, and the resulting overlap between individuals with and without MASLD may reduce the diagnostic performance of surrogate markers [12,15,16,19,20]. Moreover, pubertal development, age, sex, and degree of adiposity can influence insulin sensitivity and the interpretation of fasting insulin-based indices in adolescents [11,12,15,16,17,18,19,20,27]. Accordingly, the present findings should not be interpreted as evidence that insulin resistance is unimportant in MASLD pathophysiology. Rather, they indicate that the evaluated indices did not reliably distinguish MASLD from obesity without steatosis in this cohort. An important finding was the presence of differences in cardiac structural parameters during adolescence. LVMi and RWT were higher in both adolescents with MASLD and those with obesity without hepatic steatosis than in healthy controls. In contrast, carotid intima-media thickness (cIMT) did not differ significantly across groups. This pattern suggests that differences in cardiac structure may be detectable even when measurable differences in carotid arterial morphology are absent. Previous pediatric studies have described associations between obesity and early cardiac remodeling, potentially related to increased hemodynamic load, altered body composition, inflammation, and neurohormonal pathways [7,9,10,11,24,26,28]. These mechanisms were not directly investigated in the present study and should therefore be considered potential explanations for the observed findings. Pulse wave velocity (PWV) was higher in adolescents with MASLD than in healthy controls and adolescents with obesity without steatosis, suggesting a possible difference in arterial stiffness. However, PWV is influenced by blood pressure and other physiological factors, and the observed group differences should be interpreted cautiously. Central aortic blood pressure also differed between the MASLD and control groups, whereas peripheral blood pressure did not differ significantly across groups. Other vascular measures, including cIMT, were comparable. The results therefore suggest that vascular findings were not uniform across parameters: differences were observed in selected functional measures, while evidence of structural carotid alteration was not demonstrated. This interpretation is consistent with pediatric literature describing subclinical cardiovascular abnormalities in obesity and MASLD, although the pattern and timing of these abnormalities vary between populations and studies [9,10,24,26,28]. The regression analyses provide further information about factors associated with LVMi. Waist circumference SDS was the strongest independent correlate retained in the final multivariable model. Total cholesterol was positively associated with LVMi, whereas HDL-C showed an inverse association. In univariable analyses, several other measures of adiposity, including BMI SDS, lean body mass, fat mass, and body fat percentage, were also associated with LVMi. Because these measures are interrelated, their individual associations should not be interpreted as evidence of separate or additive effects. The selection of waist circumference SDS as a representative measure of central adiposity provides a clinically interpretable way to characterize the association between body habitus and cardiac structure, provided that collinearity and model diagnostics are adequately documented. Central adiposity may be associated with cardiac remodeling through several pathways, including increased hemodynamic demand, insulin resistance, inflammation, oxidative stress, and neurohormonal activation, as proposed in the previous literature [7,28]. However, these pathways were not directly assessed in the present study. Furthermore, the regression findings should not be interpreted as demonstrating that hepatic steatosis has no independent association with LVMi unless MASLD status was explicitly evaluated in an appropriate multivariable model. The present results more directly support an association between central adiposity and LVMi, together with additional associations involving lipid parameters, in adolescents with different obesity-related hepatic phenotypes. The ROC analyses showed poor discriminatory performance for all evaluated insulin resistance-related indices when distinguishing adolescents with MASLD from those with obesity without hepatic steatosis. AUC values ranged from 0.501 to 0.569, with HOMA-IR yielding the highest value, followed by VAI, TyG, TyG-BMI, and TyG-WC. These values indicate limited discrimination in the present cohort and do not support the use of these indices as stand-alone diagnostic tools for identifying MASLD in adolescents with obesity. Several factors may contribute to this limited performance. First, insulin resistance and metabolic abnormalities are common across the spectrum of pediatric obesity, irrespective of the presence of hepatic steatosis, potentially resulting in substantial overlap between groups [12,15,16,19]. Second, TyG-derived indices and other surrogate measures are influenced by the variables from which they are calculated, as well as by age, sex, pubertal stage, and adiposity [12,16,17,18,19,20,27]. Third, MASLD is heterogeneous and reflects the interaction of multiple factors, including adipose tissue dysfunction, lipid metabolism, genetic susceptibility, hormonal changes, and inflammatory and gut–liver pathways [7,10,29,30]. A single surrogate index is therefore unlikely to capture all relevant aspects of hepatic steatosis. Finally, physiological changes in insulin sensitivity during puberty complicate the interpretation of fasting insulin-based indices and reinforce the need for caution when applying adult-derived thresholds to pediatric populations [11,12,15]. These findings suggest that surrogate indices of insulin resistance should not be used in isolation to identify hepatic steatosis in adolescents with obesity. A broader assessment incorporating anthropometry, body composition, lipid parameters, liver biochemistry, and appropriate liver imaging may provide a more informative characterization of cardiometabolic status [9,10,11,26]. CAP measurement during transient elastography may help assess hepatic steatosis, while echocardiographic assessment may be considered when clinically indicated to evaluate cardiac structure and function. The present study does not establish a validated screening algorithm or demonstrate that a particular combination of measurements improves clinical outcomes; prospective studies are needed to assess the diagnostic and prognostic value of such approaches.

The findings indicate that adolescents with MASLD may exhibit several concurrent cardiometabolic abnormalities, including dyslipidemia, elevated liver enzyme activities, and differences in selected cardiovascular parameters. These findings support the importance of assessing hepatic and cardiovascular health in the context of pediatric obesity rather than relying exclusively on BMI or surrogate indices of insulin resistance. Early recognition of adverse metabolic profiles may provide opportunities for timely lifestyle interventions and appropriate clinical follow-up, although the optimal screening strategy and its effectiveness require prospective evaluation [9,10,11,24,26].

## 5. Conclusions

In conclusion, adolescents with MASLD exhibited a less favorable cardiometabolic profile than healthy controls, including greater hepatic steatosis, dyslipidemia, and differences in selected cardiovascular parameters. Compared with adolescents with obesity without hepatic steatosis, those with MASLD also showed differences in adiposity and selected biochemical measures. Waist circumference SDS was an important independent correlate of left ventricular mass index, whereas surrogate indices of insulin resistance showed poor discriminatory performance for distinguishing MASLD from obesity without hepatic steatosis. These findings highlight the importance of assessing hepatic and cardiovascular characteristics within the broader context of pediatric obesity. An integrated evaluation incorporating anthropometric measurements, lipid profile, liver biochemistry, and appropriate liver imaging may provide complementary information on cardiometabolic status. Prospective studies in larger pediatric cohorts are needed to determine the clinical utility of these measures and establish effective strategies for early identification and prevention of long-term metabolic and cardiovascular complications.

## 6. Strengths and Limitations

A major strength of this study is the integrated assessment of anthropometric characteristics, body composition, hepatic steatosis, biochemical parameters, vascular measures, cardiac structure, and multiple surrogate indices of insulin resistance in a well-characterized adolescent cohort. The inclusion of adolescents with MASLD, adolescents with obesity without hepatic steatosis, and healthy controls enabled comparison of cardiometabolic characteristics associated with obesity and those observed in the presence of hepatic steatosis. Several limitations should be acknowledged. First, the cross-sectional design precludes causal inference and does not allow assessment of temporal relationships between obesity, hepatic steatosis, and cardiovascular alterations. Longitudinal studies are needed to determine how these characteristics evolve over time and whether early interventions modify their trajectories. Second, the relatively small sample size may have limited statistical power to detect modest differences and associations, particularly in subgroup analyses. Sex-stratified findings should therefore be interpreted cautiously, as the estimates may be imprecise. Third, pubertal maturation, assessed using Tanner staging, was not systematically evaluated. Because puberty is associated with physiological changes in insulin sensitivity, body composition, lipid metabolism, and cardiovascular parameters, its potential influence on the observed associations could not be assessed. Fourth, lifestyle-related factors, including dietary habits and physical activity, were not systematically measured and may have contributed to differences in adiposity and cardiometabolic characteristics. Fifth, hepatic steatosis was classified primarily using the controlled attenuation parameter (CAP) measured by vibration-controlled transient elastography rather than liver histology. Although CAP provides a noninvasive assessment of hepatic steatosis, it does not provide histological information on steatohepatitis or fibrosis. In addition, liver stiffness measurements may not exclude early fibrosis. The availability of elastography measurements was not uniform across all participants, particularly in the control group, which should be considered when interpreting group comparisons involving liver stiffness. Finally, the TyG index, TyG-derived indices, and visceral adiposity index (VAI) were evaluated as surrogate markers rather than direct measurements of insulin resistance or visceral adiposity. These indices have largely been developed or studied in adult populations, and their diagnostic performance and clinically relevant thresholds in adolescents remain uncertain. Their limited discriminatory performance in the present cohort should therefore be interpreted as a finding specific to the study population and design, rather than as evidence that these indices lack clinical utility in all pediatric settings. Future prospective studies involving larger and more diverse pediatric cohorts, standardized assessment of pubertal maturation and lifestyle factors, and longitudinal evaluation of hepatic and cardiovascular outcomes are needed to validate these findings and clarify the clinical utility of surrogate metabolic indices in adolescents.

## Figures and Tables

**Table 1:** Anthropometric characteristics and body composition parameters across groups.

Variable	MASLD, Mean ± SD	Obesity Without Steatosis, Mean ± SD	Controls, Mean ± SD	Overall *p*	BH-Adjusted *p*
Age (years)	15.64 ± 1.60	15.10 ± 1.69	15.25 ± 1.67	0.170	0.184
Body weight (kg)	99.02 ± 17.45	87.96 ± 18.22	60.49 ± 12.43	7.31 × 10^−14^	1.36 × 10^−13^
Body weight SDS	2.44 ± 0.71	2.11 ± 0.62	0.16 ± 0.99	8.77 × 10^−14^	1.42 × 10^−13^
Height (cm)	173.83 ± 10.28	168.92 ± 11.00	171.83 ± 11.45	0.111	0.132
Height SDS	0.51 ± 1.16	0.21 ± 1.08	0.48 ± 1.18	0.330	0.330
BMI (kg/m^2^)	32.89 ± 5.87	30.64 ± 4.66	20.28 ± 2.40	9.58 × 10^−15^	6.13 × 10^−14^
BMI z-score (IOTF)	2.64 ± 0.58	2.45 ± 0.53	0.20 ± 1.01	1.89 × 10^−14^	6.13 × 10^−14^
BMI SDS	2.35 ± 0.56	2.26 ± 0.67	−0.03 ± 0.96	2.63 × 10^−14^	6.84 × 10^−14^
Waist circumference (cm)	98.06 ± 10.15	91.11 ± 10.27	69.36 ± 6.45	8.00 × 10^−15^	6.13 × 10^−14^
Waist circumference SDS	2.47 ± 0.88	2.03 ± 0.54	−0.28 ± 1.08	1.59 × 10^−14^	6.13 × 10^−14^
Lean body mass (kg)	64.61 ± 9.77	58.90 ± 11.50	50.87 ± 10.13	4.41 × 10^−6^	5.73 × 10^−6^
Fat mass (kg)	33.98 ± 10.80	29.58 ± 9.97	9.62 ± 4.47	3.50 × 10^−14^	7.58 × 10^−14^
Body fat (%)	33.92 ± 5.94	32.96 ± 5.68	15.55 ± 6.02	2.81 × 10^−13^	4.06 × 10^−13^

Values are presented as mean ± standard deviation (SD). The number of available observations varied by variable because of missing data. Overall group comparisons were performed using the Kruskal–Wallis test. *p*-values were adjusted for multiple testing using the Benjamini–Hochberg (BH) procedure across the 13 omnibus tests. Statistical significance was defined as a two-sided *p*-value < 0.05. Abbreviations: MASLD, metabolic dysfunction-associated steatotic liver disease; BMI, body mass index; IOTF, International Obesity Task Force; SDS, standard deviation score; SD, standard deviation.

**Table 2:** Anthropometric characteristics of female participants with MASLD, obesity, and healthy controls.

Parameter	MASLD, Mean ± SD	Obesity, Mean ± SD	Controls, Mean ± SD	Overall *p*
Age (years)	15.21 ± 1.45 ^a^	15.37 ± 2.05 ^a^	15.20 ± 1.70 ^a^	0.209
Body weight (kg)	95.42 ± 11.22 ^b^	80.75 ± 11.96 ^a^	54.23 ± 12.27 ^c^	0.001
Body weight SDS	2.60 ± 0.42 ^b^	2.05 ± 0.46 ^a^	0.03 ± 1.00 ^c^	0.001
Height (cm)	169.19 ± 8.27 ^a^	164.19 ± 11.53 ^a^	166.73 ± 7.87 ^a^	0.510
Height SDS	0.41 ± 1.24 ^a^	0.07 ± 1.00 ^a^	0.69 ± 0.75 ^a^	0.454
BMI (kg/m^2^)	33.47 ± 4.52 ^a^	29.88 ± 2.50 ^a^	19.31 ± 2.98 ^b^	0.001
BMI z-score (IOTF)	2.70 ± 0.48 ^a^	2.33 ± 0.36 ^a^	−0.27 ± 0.95 ^b^	0.001
BMI SDS (national reference)	2.53 ± 0.47 ^a^	2.39 ± 0.84 ^a^	−0.36 ± 0.94 ^b^	0.001

Values are presented as mean ± SD. Different superscript letters indicate statistically significant differences between groups based on post hoc pairwise comparisons; groups sharing at least one superscript letter are not significantly different. The overall *p*-value refers to the omnibus group comparison. The post hoc method and any adjustment for multiple comparisons is described in Section 2.8. Abbreviations: BMI, body mass index; IOTF, International Obesity Task Force; SDS, standard deviation score; MASLD, metabolic dysfunction-associated steatotic liver disease; SD, standard deviation.

**Table 3:** Anthropometric characteristics of male participants with MASLD, obesity, and healthy controls.

Parameter	MASLD, Mean ± SD	Obesity, Mean ± SD	Controls, Mean ± SD	Overall *p*
Age (years)	15.73 ± 1.65 ^a^	14.88 ± 1.47 ^a^	15.28 ± 1.57 ^a^	0.207
Body weight (kg)	99.55 ± 18.54 ^a^	84.80 ± 11.85 ^c^	64.31 ± 11.24 ^b^	<0.001
Body weight SDS	2.39 ± 0.77 ^a^	1.98 ± 0.43 ^c^	0.31 ± 0.85 ^b^	<0.001
Height (cm)	175.23 ± 10.72 ^a^	168.76 ± 8.48 ^b^	173.56 ± 11.18 ^ab^	0.043
Height SDS	0.62 ± 1.23 ^a^	0.12 ± 0.84 ^a^	0.26 ± 1.22 ^a^	0.338
BMI (kg/m^2^)	32.60 ± 6.42 ^a^	29.91 ± 5.00 ^a^	21.17 ± 1.87 ^b^	<0.001
BMI z-score (IOTF)	2.60 ± 0.64 ^a^	2.39 ± 0.51 ^a^	0.62 ± 0.62 ^b^	<0.001
BMI SDS	2.28 ± 0.63 ^a^	2.15 ± 0.63 ^a^	0.31 ± 0.59 ^b^	<0.001
Waist circumference (cm)	98.39 ± 10.42 ^a^	89.33 ± 10.46 ^c^	72.46 ± 4.28 ^b^	<0.001
Waist circumference SDS	2.47 ± 0.98 ^a^	1.85 ± 0.62 ^c^	0.05 ± 0.72 ^b^	<0.001
Lean body mass (kg)	65.77 ± 9.75 ^a^	57.42 ± 7.37 ^b^	55.08 ± 8.58 ^b^	<0.001
Fat mass (kg)	33.25 ± 12.01 ^a^	26.49 ± 7.78 ^c^	9.23 ± 3.53 ^b^	<0.001
Body fat (%)	32.82 ± 6.54 ^a^	31.30 ± 5.46 ^a^	14.00 ± 3.67 ^b^	<0.001

Values are presented as mean ± SD. Different superscript letters indicate statistically significant differences between groups based on post hoc pairwise comparisons; groups sharing at least one superscript letter are not significantly different. The overall *p*-value refers to the omnibus group comparison. The post hoc method and any adjustment for multiple comparisons is described in Section 2.8. Abbreviations: BMI, body mass index; IOTF, International Obesity Task Force; SDS, standard deviation score; MASLD, metabolic dysfunction-associated steatotic liver disease; SD, standard deviation.

**Table 4:** Biochemical and elastography parameters across groups (median, IQR).

Variable	MASLD Median (Q1–Q3)	Obesity Without Steatosis Median (Q1–Q3)	Controls Median (Q1–Q3)	Overall *p*	Overall *p* (BH)	*n* MASLD	*n* Obesity	*n* Controls
Liver stiffness, E [kPa]	5.2 (4.5–7.2)	5.1 (4.6–5.6)	5.4 (4.2–6.1)	0.8181	0.8181	49	22	22
CAP [dB/m]	293.0 (273.0–315.0)	222.0 (205.0–233.5)	189.0 (176.0–205.0)	<0.0001	<0.0001	49	22	21
ALT [U/L]	33.5 (21.5–46.5)	20.0 (14.75–32.5)	12.0 (9.8–13.0)	<0.0001	<0.0001	46	28	24
AST [U/L]	23.0 (18.0–32.0)	20.0 (17.0–24.5)	14.5 (12.0–19.0)	<0.0001	0.0001	45	27	24
Total bilirubin [mg/dL]	0.560 (0.405–0.732)	0.715 (0.585–0.900)	0.660 (0.490–1.960)	0.1091	0.1510	42	26	23
CRP [mg/dL]	0.200 (0.100–0.300)	0.230 (0.100–0.400)	0.100 (0.100–0.175)	0.3454	0.4145	36	13	14
GGT [U/L]	23.0 (16.5–33.0)	16.0 (12.0–24.5)	16.0 (12.0–22.0)	0.0039	0.0059	43	27	21
Total cholesterol [mg/dL]	158.5 (138.5–177.5)	153.0 (131.0–168.5)	157.0 (133.0–171.0)	0.5695	0.6407	46	27	25
Triglycerides [mg/dL]	126.0 (91.3–176.3)	92.0 (70.0–121.5)	66.5 (54.3–78.0)	<0.0001	<0.0001	46	27	26
LDL-C [mg/dL]	89.0 (70.0–105.8)	95.2 (69.8–111.0)	94.0 (63.6–106.5)	0.7513	0.7955	45	25	23
HDL-C [mg/dL]	40.0 (36.0–45.0)	41.0 (38.0–44.0)	54.0 (49.0–60.0)	<0.0001	<0.0001	46	25	23
Glucose [mg/dL]	89.3 (85.7–94.4)	89.2 (83.7–92.9)	87.1 (85.6–89.5)	0.2523	0.3244	44	22	26
Insulin [µIU/mL]	15.3 (10.4–21.9)	11.95 (9.8–15.8)	9.4 (8.3–13.5)	0.0015	0.0024	47	26	24
HOMA-IR	3.40 (2.6–4.9)	2.84 (2.1–3.8)	2.01 (1.8–2.8)	0.0004	0.0007	44	21	24
TyG index	8.68 (8.3–9.0)	8.40 (8.1–8.7)	7.95 (7.8–8.0)	<0.0001	<0.0001	44	20	24
VAI	1.94 (1.4–2.7)	1.51 (0.9–1.7)	0.75 (0.6–0.9)	<0.0001	<0.0001	45	25	23
TyG-BMI	271.5 (242.2–305.0)	236.9 (223.9–275.9)	156.4 (152.0–169.3)	<0.0001	<0.0001	44	20	24
TyG-WC	826.5 (757.1–911.1)	742.2 (668.7–828.9)	553.7 (508.8–584.2)	<0.0001	<0.0001	43	20	24

Values are presented as median (interquartile range). Group comparisons were performed using the Kruskal–Wallis test followed by Dunn’s post hoc test. The Benjamini–Hochberg procedure was applied across the 18 omnibus group comparisons. The overall *p*-value refers to the omnibus test; BH-adjusted *p*-values are reported alongside nominal values. Statistical significance was defined as a two-sided *p* < 0.05. One control participant had liver stiffness measured without concurrent CAP measurement due to technical limitations during the examination. Abbreviations: ALT, alanine aminotransferase; AST, aspartate aminotransferase; BH, Benjamini–Hochberg; CAP, controlled attenuation parameter; CRP, C-reactive protein; E, liver stiffness; GGT, gamma-glutamyl transferase; HDL-C, high-density lipoprotein cholesterol; HOMA-IR, homeostatic model assessment for insulin resistance; IQR, interquartile range; LDL-C, low-density lipoprotein cholesterol; MASLD, metabolic dysfunction-associated steatotic liver disease; TyG, triglyceride–glucose index; TyG-BMI, triglyceride–glucose index × body mass index; TyG-WC, triglyceride–glucose index × waist circumference; VAI, visceral adiposity index.

**Table 5:** Vascular and cardiovascular parameters across study groups.

Variable	MASLD (*n* = 50)	Obesity (*n* = 33)	Controls (*n* = 28)	Overall *p*-Value
PWV (m/s)	5.70 (5.18–6.10) ^b^	5.25 (4.90–5.50) ^a^	5.00 (4.80–5.60) ^a^	0.030
Systolic BP (mmHg)	120 (112–129) ^a^	115 (110–125) ^a^	114 (110–124) ^a^	0.290
Diastolic BP (mmHg)	72 (64–77) ^a^	73 (65–79) ^a^	68.5 (61–71) ^a^	0.165
95th percentile AoBP (mmHg)	119 (115–124) ^a^	115.5 (114.5–122) ^a^	115 (114–119) ^a^	0.235
Aortic BP (AoBP) (mmHg)	113 (109–123) ^b^	109.5 (107.5–117.5) ^a^	107.5 (104–116) ^a^	0.049
AoBP > 95th percentile (%)	0 (0–1) ^a^	0 (0–0) ^a^	0 (0–0) ^a^	0.266
IMTR (mm)	0.44 (0.43–0.47) ^a^	0.43 (0.40–0.47) ^a^	0.44 (0.42–0.45) ^a^	0.318
IMTL (mm)	0.44 (0.41–0.48) ^a^	0.43 (0.41–0.46) ^a^	0.42 (0.42–0.45) ^a^	0.493
cIMT (mm)	0.44 (0.42–0.48) ^a^	0.43 (0.41–0.47) ^a^	0.44 (0.42–0.45) ^a^	0.371
WCSA (mm^2^)	7.89 (7.45–8.48) ^b^	7.47 (6.93–8.76) ^a^	7.03 (6.62–7.65) ^a^	0.002
LVMi (g/m^2^)	38.09 (33.22–41.54) ^a^	35.54 (30.70–40.40) ^a^	26.59 (21.51–30.12) ^b^	<0.001
RWT	0.36 (0.31–0.40) ^a^	0.35 (0.34–0.37) ^a^	0.31 (0.30–0.34) ^b^	0.008

Values are presented as median (interquartile range). Different superscript letters indicate statistically significant differences between groups based on post hoc pairwise comparisons; groups sharing at least one superscript letter are not significantly different. The overall *p*-value was obtained from the omnibus test. Statistical significance was defined as *p* < 0.05. Abbreviations: AoBP, aortic blood pressure; BP, blood pressure; cIMT, common carotid intima-media thickness; IMTL, intima-media thickness of the left carotid artery; IMTR, intima-media thickness of the right carotid artery; LVMi, left ventricular mass index; MASLD, metabolic dysfunction-associated steatotic liver disease; PWV, pulse wave velocity; RWT, relative wall thickness; WCSA, wall cross-sectional area.

**Table 6:** Sex-specific cardiovascular and vascular parameters.

**(A) females**				
Variable	MASLD (*n* = 9)	Obesity (*n* = 15)	Controls (*n* = 9)	Overall *p*-Value
PWV (m/s)	5.60 (5.10–6.00) ^a^	5.20 (4.90–5.60) ^a^	5.10 (4.80–5.40) ^a^	NS
Systolic BP (mmHg)	118 (110–126) ^a^	116 (110–124) ^a^	114 (110–120) ^a^	NS
Diastolic BP (mmHg)	71 (64–76) ^a^	73 (65–78) ^a^	68 (61–71) ^a^	NS
Aortic BP (AoBP) (mmHg)	112 (108–120) ^a^	110 (107–118) ^a^	107 (104–114) ^a^	NS
cIMT (mm)	0.44 (0.42–0.47) ^a^	0.43 (0.41–0.46) ^a^	0.44 (0.42–0.45) ^a^	NS
WCSA (mm^2^)	7.8 (7.4–8.4) ^a^	7.5 (6.9–8.2) ^a^	7.0 (6.6–7.6) ^a^	NS
LVMi (g/m^2^)	38.7 ± 7.1 ^a^	35.8 ± 6.8 ^a^	27.9 ± 5.6 ^b^	0.020
RWT	0.36 ± 0.04 ^a^	0.35 ± 0.03 ^a^	0.32 ± 0.03 ^a^	NS
**(B) males**				
Variable	MASLD (*n* = 41)	Obesity (*n* = 18)	Controls (*n* = 19)	Overall *p*-Value
PWV (m/s)	5.70 (5.18–6.10) ^a^	5.25 (4.90–5.50) ^ab^	5.00 (4.80–5.60) ^b^	0.030
Systolic BP (mmHg)	120 (112–129) ^a^	115 (110–125) ^a^	114 (110–124) ^a^	NS
Diastolic BP (mmHg)	72 (64–77) ^a^	73 (65–79) ^a^	68 (61–71) ^a^	NS
Aortic BP (AoBP) (mmHg)	113 (109–123) ^a^	109 (107–118) ^ab^	107 (104–116) ^b^	0.049
cIMT (mm)	0.44 (0.42–0.48) ^a^	0.43 (0.40–0.47) ^a^	0.44 (0.42–0.45) ^a^	NS
WCSA (mm^2^)	7.89 (7.45–8.48) ^a^	7.47 (6.93–8.76) ^ab^	7.03 (6.62–7.65) ^b^	0.002
LVMi (g/m^2^)	38.1 ± 7.5 ^a^	35.5 ± 7.2 ^a^	27.2 ± 5.8 ^b^	<0.001
RWT	0.36 ± 0.05 ^a^	0.35 ± 0.04 ^ab^	0.32 ± 0.03 ^b^	0.003

Values are presented as median (interquartile range) or mean ± standard deviation, as appropriate. Different superscript letters indicate statistically significant differences between groups based on post hoc pairwise comparisons; groups sharing at least one superscript letter are not significantly different. NS, not significant; statistical significance was defined as *p* < 0.05. Abbreviations: AoBP, aortic blood pressure; BP, blood pressure; cIMT, carotid intima-media thickness; LVMi, left ventricular mass index; NS, not significant; PWV, pulse wave velocity; RWT, relative wall thickness; WCSA, wall cross-sectional area.

**Table 7:** Correlations between insulin resistance indices and anthropometric, metabolic, hepatic, and inflammatory parameters.

Variable	HOMA-IR	TyG	VAI	TyG-BMI	TyG-WC
Age	0.061 (0.596)	0.172 (0.135)	0.184 (0.107)	0.149 (0.196)	0.225 (0.051)
Weight	0.471 (<0.001)	0.566 (<0.001)	0.400 (<0.001)	0.883 (<0.001)	0.875 (<0.001)
Height	0.227 (0.047)	0.277 (0.015)	0.123 (0.284)	0.032 (0.780)	0.223 (0.053)
BMI	0.385 (<0.001)	0.470 (<0.001)	0.354 (0.001)	0.972 (<0.001)	0.862 (<0.001)
Waist circumference	0.410 (<0.001)	0.606 (<0.001)	0.406 (<0.001)	0.930 (<0.001)	0.963 (<0.001)
Fat mass	0.448 (<0.001)	0.518 (<0.001)	0.420 (<0.001)	0.952 (<0.001)	0.861 (<0.001)
Lean body mass	0.445 (<0.001)	0.502 (<0.001)	0.263 (0.024)	0.636 (<0.001)	0.706 (<0.001)

Values are Pearson correlation coefficients (r) with corresponding nominal *p* values in parentheses. Correlations with nominal *p* < 0.001 remained significant after Benjamini–Hochberg adjustment. Abbreviations: BMI, body mass index; HOMA-IR, homeostatic model assessment for insulin resistance; TyG, triglyceride–glucose index; TyG-BMI, triglyceride–glucose index × body mass index; TyG-WC, triglyceride–glucose index × waist circumference; VAI, visceral adiposity index.

**Table 8:** Univariate and multivariable regression analyses of factors associated with left ventricular mass index (LVMi).

Predictor	Univariable β	*p*-Value	Multivariable β	*p*-Value
BMI SDS	28.80	<0.0001	—	—
Waist circumference SDS	27.23	<0.0001	21.37	<0.0001
Lean body mass (kg)	2.87	<0.0001	—	—
Body fat mass (kg)	2.87	<0.0001	—	—
Body fat (%)	3.16	<0.0001	—	—
Total cholesterol (mg/dL)	0.41	0.0318	0.45	0.0046
Triglycerides (mg/dL)	0.44	<0.0001	—	—
HDL-C (mg/dL)	−2.44	0.0001	−1.74	0.0030
HOMA-IR	9.29	0.020	—	—
TyG index	0.010	<0.0001	—	—
TyG-BMI	0.260	0.0002	—	—

β, regression coefficient. Univariable β coefficients represent the change in LVMi associated with a one-unit increase in the respective predictor. Multivariable β coefficients represent the independent associations with LVMi after adjustment for the variables retained in the final multivariable model. A dash (—) indicates that the variable was not retained in the final multivariable model, in accordance with the predefined inclusion criteria described in Section 2.8. *p* < 0.05 was considered statistically significant.

## Data Availability

The data are available upon request.
